# Dementia with Parkinson disease: Clinical diagnosis, neuropsychological aspects and treatment

**DOI:** 10.1590/S1980-57642009DN20400005

**Published:** 2008

**Authors:** Jorge Lorenzo Otero

**Affiliations:** Profesor Agregado, Director del Departamento de Neuropsicología, Instituto de Neurología Facultad de Medicina, Montevideo, Uruguay.

**Keywords:** dementia with Parkinson’s disease, Parkinson’s disease, cognitive impairment, Alzheimer’s disease, Lewy body dementia, mild cognitive impairment

## Abstract

Dementia with Parkinson’s disease represents a controversial issue in the complex group of alpha-synucleinopathies. The author acknowledges the concept of a “continuum” between Parkinson disease’s (PD), Lewy body dementia (LBD), and dementia in Parkinson’s disease (PDD). However, the practicing neurologist needs to identify the phenotypic signs of each dementia. The treatment and prognosis are different in spite of the overlaps between them. The main aim of this review was to characterize the clinical diagnoses of dementia associated with Parkinson’s disease (PDD). Secondarily, the review discussed some epidemiological and neuropsychological issues. Selection of articles was not systematic and reflects the author’s opinion, where the main text selected was the recommendations from the Movement Disorder Society Task Force for PDD diagnosis. The Pub Med, OVID, and Proquest data bases were used for the search.

## Abstract (pt)

Demência com doença de Parkinson constitui um assunto controverso no complexo grupo das alfa-sinucleinopatias. O autor admite o conceito de um “continuum” entre doença de Parkinson (DP), demência com corpos de Lewy (DCL) e demência na doença de Parkinson (DDP). Todavia, neurologistas necessitam identificar os sinais fenotípicos de cada uma. O tratamento e prognóstico são diferentes, apesar das sobreposições entre elas. O primeiro objetivo desta revisão foi o de caracterizar o diagnóstico clínico de demência associada à DP. Secundariamente, a revisão discute alguns aspectos epidemiológicos e neuropsicológicos. A seleção dos artigos não foi sistemática e reflete a opinião do autor, escolhendo como texto principal as recomendações da Força Tarefa da Sociedade de Transtornos do Movimento para diagnóstico de DDP. Foram utilizados as bases de dados do Pub Med, OVID e Proquest como bases de dados.

Neurodegenerative diseases are frequently associated with intellectual deterioration. In recent years, advances in our knowledge of their neuropathology allow them to be classified, not only by their phenotypical characteristics, but also according to neuropathology and genetic substrates.

The neuropathology involves, among other causes, protein alterations which are genetically determined, and sometimes unknown environmental factors. As an example: Alzheimer’s disease (AD), the most frequent degenerative disease in aged adults, is linked with the mutation (in familial cases) of genes involved in regulation of amyloid beta and tau protein production. Genetic causes, associated with less well-known environmental factors, account for the sporadic AD in aged adults.^1^

The frontotemporal dementias are linked to genes that regulate the tau protein, known generically as tauopathies, and also with the genes that regulate the TDP-43 protein, among them the progranulin gene.^2^

Dementia with Parkinson’s disease (PDD) is included in a cluster of encephalopathies related with the mutation or polymorphism of alpha-synucleins. The alpha-synuclein mutations, discovered by Polymeropulos,^3^ related to PARK1 pathologically folded and toxic, is the central component of Lewy Bodies and the neuritis of Lewy.^4^ They compose, in the multi-systemic atrophies, the filaments of Papp-Lantos, located in the oligodendroglia and neurons.

The alpha-synucleinopathies includes:^5^

1. Parkinson’s Disease (PD).
2. Lewy Body Diseases and Dementia with Lewy Bodies. The former includes the “spectrum” of Lewy body diseases and the latter, Lewy Body Dementia (LBD).
3. Multisystem Atrophies (MA).
4. Hallervorden-Spatz disease, whose classification into the group of alpha-synucleinopathies is controversial.

## Some epidemiological aspects of cognitive impairment in PD, MCI-PD and PDD

The studies differ in many aspects. Most were carried out in hospital populations, while longitudinal “door to door” studies were uncommon.

Methodology used in the studies in specialized centers differs from those of population sample cohorts, giving rise to dissimilar results.

## Prevalence

The Rotterdam Study cohort study^6^ with a follow up of 8 years, based on a “door to door” methodology, includes milder and more moderate cases in comparison with the clinical studies, so yields lower prevalence values. Aarsland and cols found that 75% of the sample evolved to dementia according to DSM-III-R criteria. The akinetic forms and presence of hallucinations increased the risk of evolution to dementia. In addition, these patients had lower-than-expected MMSE scores for their level and education, on the cognitive evaluation of the UPDRS (motor scale for EP) and on other less well-known cognitive scales.

## Incidence

Incidence varied between studies.^7-9^ Approximately 10% of the population is estimated to develop dementia annually. Recently Butler et al.^10^ published a 12-year population follow up. By the end of the study period, 60% of patients had developed dementia.

“Women live with PD longer than men and spend more years with dementia. At age 70 years a man with PD but not dementia has a life expectancy of 8 years, 5 years of which can be expected to be dementia free, and 3 years with dementia”

## Conclusions

The prevalence rate of dementia in PDD is almost 30% while the incidence rate is four to six times higher than controls. In other words, patients with PD have a 4 to 6-fold greater chance of developing dementia than normal subjects with similar age, education and socioeconomic level.

The main risk variables are older age, gender, more severe Parkinsonism, in particular rigidity, postural instability, gait impairments and slight cognitive deterioration at disease onset.

### Cognitive impairment in early stages

Although James Parkinson´s initial description emphasized the absence of cognitive impairments in PD, cognitive impairment in PD is a well-known phenimenon,^11^ and has been systematically studied over the last thirty years. Cognitive impairment in PD has been described at time of diagnosis in approximately 24% of patients.^12^

A three-year cohort study showed cognitive alterations with predominance of executive dysfunction in parkinsonian patients, which did not evolve to dementia in 54% of cases.^13^ Differences are explained by methodological reasons, time of evolution of the disease and patient characteristics.

### From early stage to mild cognitive impairment and PDD

Janvin et al., in a cross-sectional study of 103 patients with PD, reported that 27 were demented, 42 had MCI and 34 were normal.^14^

A few authors have found heterogeneous impairments, although the majority emphasize the frequency of executive impairments in recently diagnosed patients.^13-15^

The incidence of tremors in initial stages is controversial. Vingerhoets et al.^16^ claimed that patients with initial tremor are more likely to suffer cognitive impairment in the more advanced stages of PD compared with patients with akinesia and rigidity at onset.

Although cognitive alterations are frequent in PD, they do not significantly impair activities of daily living, and so not fulfill the DSM-IV criteria for dementia.

A few authors have proposed the diagnosis of Parkinsonian Mild Cognitive Impairment.^17-18^ It would be characterized by prominent amnesic-dysexecutive alterations.

Caviness et al. concluded that a stage of clinical cognitive impairment in PD exists between the stages of PD without cognitive impairment and PD with dementia, using DSM-IV criteria for dementia^19^ where this stage may be defined by applying criteria similar to that of amnestic mild cognitive impairment (MCI), which is proposed as a precursor of AD.

Recently, GEPAD,^20^ a German nationwide, epidemiological and cross-sectional study, included a representative sample of 1449 outpatients with parkinsonian syndrome that were thoroughly assessed by 315 neurologists, each assessing up to five patients on a single study day, in September/October 2005. According to the UK Brain Bank criteria, 873 patients were diagnosed as having PD, and of these none met criteria for a certain PD diagnosis according to UK Brain Bank criteria. Results allow us to conclude:

“Almost one third of all PD outpatients suffered from dementia and more than one in five are afflicted by depression. Neuropsychiatric further syndromes contribute to the risk of dementia. The prevalence estimates of cognitive impairment vary considerably, depending on the diagnostic measure used. There is evidence in our results that the standard screening tool for dementia (MMSE) is inappropriate for PD patients due to its lack of sensitivity (50%)”.

## Discussion

Quality of life in parkinsonian patients depends on all stages of a number of factors. Carod-Artal et al. studied a Brazilian cohort of PD patients concluding that main Health Related Quality of Life determinants in these patients were mood disorders, disability, PD complications, and years of education.^21^

In early PD stages, approximately 30% of the patients manifest minimal cognitive impairment in memory and executive function. A sub-group of patients including the 30% showing cognitive impairment at baseline, goes on to develop MCI of the amnesic-dysexecutive type, which is considered a multiple domain MCI. These patients have high risk of evolving to Dementia.

Definition of Dementia according to the DSM-IV criteria, as well as the poor sensitivity of several of cognitive screening tests (for example, the MMSE), gives rise to very variable rates of prevalence and difficulties in the concept definition of PD-MCI and PDD. Barriers to reaching a consensus on PDD are associated are attributed to a number of reasons. Diagnostic criteria for dementia may be a useful model for AD but are insufficient for the diagnosis of other dementias, including LBD and PDD. Consequently, the lll Report of the Lewy Body Disease Consortium has called for a more precise working definition of PDD that allows positive and differential diagnosis between both clinical entities.

Therefore, in response to this recommendation, the Movement Disorders Society created a Work group for PDD diagnosis consensus.

We shall now dedicate our attention to PDD, reviewing and debating the diagnosis consensus.

### PDD Diagnosis

The “core features” described in the Clinical Criteria Diagnosis of Dementia associated with Parkinson’s disease^22^ characterize a dementia syndrome with insidious onset and slow progression, developing within the context of established PD, diagnosed according to the Queen Square Brain Bank criteria and confirmed by history, clinical, and mental examination, defined as:

- Impairment in more than one cognitive domain
- Representing a decline from premorbid level
- Deficits severe enough to compromise daily life (social, occupational, or personal care), independent of the impairment ascribable to motor or autonomic symptoms.

The Movement Disorder Society Task Force has established practical criteria for diagnosis of probable and possible PDD.^23^ They determine two levels of certainty:

Level I, for use by the clinical practitioner:

Level II establishes neuropsychological norms and specific tests for use in clinical studies or tests with drugs or for those cases in which the procedures of Level I are insufficient for a reliable diagnosis.

In Level I, the cognitive disorders are determined from a factorial analysis of the sub-items of the MMSE,^24^ the Clock Drawing Test^25^ and/or Word Recall and Lexical Fluency^26^ with established cut-off scores.

Level II requires a complete group of batteries and neuropsychological tests of cognitive disorders, including behavioral impairments, in those patients with severe motor limitations, where the author deemed the ability to self-administer medication as a criterion of autonomy in PDD.

The clinical diagnosis of PDD remains controversial.

The concept of a pathological “continuum” in the alpha-synucleinopathies with different degrees of lesion and distribution of Lewy lesions often hamper phenotype identification and confounds the clinician in diagnosing LBD or PDD.

Development of PDD is correlated with the following factors:^27^

1. Age greater then 70 years.
2. UPDRS greater than to 25 (scale with three subsections) 1–mental, conduct and mood; 2–Activities of daily life; 3–motor function). A score higher than 25 implies moderate impairment.
3. Depression.
4. Visual hallucinations: Aarsland in PD found hallu­cinations in 14% of PD patients, 54% of PDD and 76% of LBD cases.^6^
5. Odd habit, agitation, disorientation or psychosis with L- Dopa.
6. Psychological exhibition to stress.
7. Presence of cardiovascular anomalies.
8. Low socioeconomic level.
9. Postural bradykinesia and gait impairment.
10. Parkinsonian tremor or other signs not associated with dementia.
11. The presence of dementia at Disease onset does not sustain the diagnosis of PDD, but Dementia with parkinsonian features.
12. Patients with PDD exhibit a wide variety of cognitive alterations ranging from impairment in several specific cognitive domains to severe dementia. The presence of isolated cognitive disorders is not a sign of Dementia, on the contrary, PDD manifests in the course of at least a year (often 10 or more) after a well-established typical PD.

The onset of dementia in the course of PD often forces institutionalization of the patient.

The differential diagnosis with LBD remains unclear. The rule of one year of dementia prior to developing parkinsonian signs in LBD (not easy in clinical practice) remains the key differential criteria.

### Neuropsychological aspects

The Work Group of the Society of Abnormal Movements^22^ concluded the following with regard to cognitive functions in PD and AD:

### Attention

One of the investigations focusing on attention, found moderate impairment in terms of variability of yields over time, based on a series of tests of reaction time.^28^ These showed increase in variability in both groups of patients with PDD and LBD versus controls and patients with AD. Clinically, 29% of the patients with PDD showed evidence of attention fluctuations compared with 42% of the patients with LBD. It was possible to conclude that attention is much more affected in PDD than in AD, and that it can fluctuate. LBD however, has more attentional disorders and more daily fluctuations. Periodic cognitive oscillations in daily performance are one of the core criteria for LBD diagnosis.^29^

#### Memory

It can be concluded that verbal memories are more affected than visual ones, and degree of impairment degree is perhaps less than that seen in AD, while recognition can be less affected than evocation, particularly in the cases of severe or moderate PDD. Implicit memory is relatively preserved both in PDD and AD, and akin to PD, clues can improve performance.^30,31^

#### Executive function

The results of the reviewed studies reveal that the executive function is affected more in patients with PDD, than in patients with AD. Evidence exists that indicates the memory disorders in PDD are more closely associated with executive dysfunction than in AD.^32,33^ The tests used to evaluate executive function in PD have shown strong correlation among results, with the exception of the WCST, possibly because of the wide divergence of scores between patients.^34^

#### Language

Language function has received little attention in PDD, perhaps because clinically evident aphasia is rare. Scant available data suggest that PDD patients are less impaired in core language functions compared to AD subjects. Consensus point to progressive anomia, and impairment in the understanding of complex sentences.

There is evidence that patients with PD manifest a deficit in both action and object naming, compared with controls. In addition, patients with PD but not controls, were significantly more impaired in action than in object naming. The current study supports the view that action naming is affected in patients with PD, possibly reflecting the presence of prefrontal dysfunction.^35^

#### Construction and praxis

PDD patients manifest visuospatial and visuoconstructive impairments. The tasks that evaluate them include a strong executive element.

There are no studies showing to what extent motor impairment affects visuoconstructive tasks. Little is known about the primary source of the defects in PDD in the accomplishment of visuoconstructive tasks.

A study using^36^ the qualitative evaluation of the Clock Drawing Test showed evidence of a more severe defect in planning in PDD compared with a patient group with AD. Visuospatial construction is probably an impairment in PDD, to a greater degree than in AD, but less than in LBD.

#### Visuospatial functions

In the review article, the Group concludes that PDD is associated with substantial visuospatial disorders, similar to LBD but qualitatively different. Recently, Silva et al.^37^ revised the value of, and patient performance on a set of different classical tests for visuospatial function, concluding that moderate correlation exists between tests for evaluating visuospatial functions.

The author emphasizes that the results are restricted by the bias in the selected test of “non-visuospatial” factors such as attention and executive functions.

### Conclusions on cognitive impairment profiles

Some differences between PDD and AD exist, particularly in executive function, whereas a dysexecutive or subcortical pattern predominates in PDD.^38^

AD, LBD and PDD have different patterns of impairment, where the motor deficit in PDD precludes the measurement of activities of daily living. The differences are more evident in the early stages and difficult to identify in evolved patients. Many studies involving an broad variety of tests failed to distinguish patients with PDD, AD and LBD with any certainty. The distinction between patients with PDD and patients with LBD in cognitive terms it is even less clear.^29^ Several of the tests employed have low sensitivity for determining the alternative mechanisms involved: e.g. the role of attentional impairment in the amnestic function as opposed to the primary defects of memory.

Neuropsychological evaluation plays an important role in providing objective evidence of cognitive disorders to support the clinical diagnosis of PDD.

Nevertheless, the contribution of neuropsychology in the differential diagnosis is not conclusive, at least with the currently used tests.

The evidence is not sufficiently robust to base a diagnosis solely on standard tests.

From the cognitive and practical point of view, we believe that in patients diagnosed for the first time as PD, the neuropsychological evaluation is necessary to distinguish the group of high risk patients (Parkinsonian MCI) in the initial stages. Unlike suspected PDD cases, the clinician may follow the Consensus recommendation: use the MMSE in its different sub-items, and level 1 test, with particular emphasis on ADL (not always easy to evaluate in advanced PD). if doubts persist, a Level 2 specific neuropsychological evaluation may be needed. The Group insists on the need to establish whether the patient harboring a PDD or not by its present therapy and prognosis.^39^ Paraclinical methods, including neuroimaging, are controversial as diagnostic methods, but are promising approaches.

### Behavior and neuropsychiatric facts

We did not list the different behavioral disorders described in the bibliography and updated by the Group of Tasks of the Society of Abnormal Movements since they are not useful for nosologically distinguishing the different alpha- synucleinopathi­es.

Neuropsychiatric symptoms are common in all the types of dementia and have very little specific value toward establishing differential diagnoses. The hallucinations described by McKeith et al. in the First Consensus about Lewy Bodies Disease (1996), remains one of the few useful facts in the distinction between LBD, PDD and AD, being most prominent in LBD. In terms of facts, the patients with PDD have less frequent and intense psychotic symptoms than patients with LBD. These differences, nevertheless, may simply reflect different degrees of severity of the dementia.

The situation is similar in distinguishing subjects with PDD from others with PD, since such neuropsychiatric symptoms are frequent in parkinsonian patients without dementia.

Therefore, although it is certain that neuropsychiatric disorders can be a risk factor for the development of dementia, and can support its diagnosis, they do not seem useful when distinguishing between PDD, LBD and AD in individual cases.

However, there is no unanimous agreement with the conclusions of the PDD Task Force Report. Recently, Weintraub^40^ and colleagues claimed that neuropsychiatric symptoms such as depression, anxiety, sleep disorders and wakefulness, psychosis and dementia are more debilitating than motor impairment in PDD.

There is a significant difference in neuropsychiatric, cognitive and motor signs between LBD, PDD and AD.

## Figures and Tables

**Table:** Algorithm for diagnosing PDD at Level I (Dubois, et al.).^23^

1.	A diagnosis of Parkinson's disease based on the Queen's Square Brain Bank criteria for PD
2.	PD developed prior to the onset of dementia
3.	MMSE score below 26
4.	Cognitive deficits severe enough to impact daily living (Caregiver interview or Pill Questionnaire)
5.	Impairments on at least two of the following tests: Mini-Mental State Examination^24^ MMSE pentagons Months reversed or seven backward Word recall or clock drawing Lexical fluency
